# Severe growth faltering and persistent hyperchloremic metabolic acidosis as early clues of renal tubular acidosis in a neonate: a case report

**DOI:** 10.1093/omcr/omaf294

**Published:** 2026-02-18

**Authors:** Nafisa Mariam, Monira Sarmin, Nusrat Jahan Shaly, Mosharrat Tabassum, Shamima Sharmin Shikha, Didarul Haque Jeorge, Farzana Afroze, Lubaba Shahrin, Tahmeed Ahmed, Chowdhury Ali Kawser, Mohammod Jobayer Chisti

**Affiliations:** International Centre for Diarrhoeal Disease Research, Bangladesh (icddr,b); International Centre for Diarrhoeal Disease Research, Bangladesh (icddr,b); International Centre for Diarrhoeal Disease Research, Bangladesh (icddr,b); International Centre for Diarrhoeal Disease Research, Bangladesh (icddr,b); International Centre for Diarrhoeal Disease Research, Bangladesh (icddr,b); International Centre for Diarrhoeal Disease Research, Bangladesh (icddr,b); International Centre for Diarrhoeal Disease Research, Bangladesh (icddr,b); International Centre for Diarrhoeal Disease Research, Bangladesh (icddr,b); International Centre for Diarrhoeal Disease Research, Bangladesh (icddr,b); International Centre for Diarrhoeal Disease Research, Bangladesh (icddr,b); International Centre for Diarrhoeal Disease Research, Bangladesh (icddr,b)

**Keywords:** bicarbonate therapy, diarrhoea, failure to thrive, neonate, renal tubular acidosis, septic shock, Dhaka, Bangladesh

## Abstract

Renal tubular acidosis (RTA) often presents as failure to thrive in children. In a resource-limited country, the diagnosis of renal tubular acidosis can be delayed or even missed because of the co-existing high burden of malnutrition. Here we report a case of a neonate/infant who presented with diarrhoea and failure to thrive and subsequently developed septic shock. During the management of the child, proximal renal tubular acidosis was diagnosed based on the persistence of metabolic acidosis with hyperchloremia. Proximal renal tubular acidosis (pRTA) can lead to complications, such as electrolyte disorders, bony deformities. Prompt diagnosis, appropriate treatment, and long-term follow-up are imperative for achieving good outcomes.

## Background

Renal tubular acidosis (RTA) comprises a group of defects in the reabsorption of bicarbonate (HCO_3_^−^), the excretion of hydrogen ions (H^+^), or both in the kidneys [1]. Out of four types of RTA, Type 1 or distal RTA, Type 2 proximal RTA, and Type 4 or hyperkalaemic RTA are the major types [2]. The fourth type (Type 3 RTA) presents as a combination of features of Type 1 and 2, but is rare and sometimes classified as a subtype of distal RTA [3, 4]. Genetic studies reveal that the prevalence of renal tubular acidosis is high in South East Asia, particularly in Thailand, Malayasia, the Philippines and Papua New Giunea [5]. From India, a hospital record-based retrospective study found 66 cases of RTA between January 2016 to December 2018 of which 6 (9.1%) had proximal RTA, 58 (87.9%) had distal RTA and the rest (3%) had other types of RTA [6].

Proximal renal tubular acidosis (pRTA) can occur either as part of a generalized proximal tubular dysfunction, known as Fanconi syndrome, or as an isolated condition [7]. The most common clinical presentation is failure to thrive [8]. Polyuria, polydipsia, dehydration, vomiting, anorexia, constipation, and muscle weakness are additional features that primarily result from hypokalemia [8]. To date, few cases have been reported in Bangladesh, most of which were adults [9, 10]. However, these cases are being regularly diagnosed at different tertiary centers.

Early diagnosis of RTA is particularly challenging in neonates for a number of reasons. Firstly, newborns, particularly those born prematurely, often have underdeveloped renal tubules that may not effectively eliminate hydrogen ions or reabsorb bicarbonate. As a result, the urinary anion gap (UAG) may be poorly defined or even negative [11]. Secondly, despite improvements, malnutrition remains a major issue in Bangladesh with around 10% of children under five still suffering from wasting [12]. Malnourished children commonly present with a plethora of complications or acute infections such as diarrhea, electrolyte disorder, pneumonia, and sepsis [13]. Any of these may cause failure to thrive, thus masking the concurrent presence of RTA. Thirdly, diagnosing RTA requires blood and urine tests to check the body’s acid–base balance and look for typical electrolyte problems. It is also important to find issues in how the kidneys handle bicarbonate, hydrogen ions, and other substances. This can be challenging in settings with limited resources. For economically disadvantaged families, it is hard to afford these tests and a long hospital stay. As a result, many leave before completing the treatment.

Here, we report a child who presented with diarrhea and failure to thrive, followed by septic shock who was later diagnosed with proximal renal tubular acidosis, but was treated successfully at Dhaka Hospital of the International Centre for Diarrhoeal Disease Research, Bangladesh (icddr,b).

## Case presentation

A 27-day-old female infant from a peri-urban area of Dhaka was brought to the Dhaka Hospital of icddr,b by her parents on August 17, 2021. She presented with fever and cough for one day, and loose stool for about 8 hours. The fever was low-grade, intermittent and subsided after sponging it with lukewarm water. She had passed stool about 5–6 times, which was yellowish, devoid of blood or mucus, not associated with straining. She was not administered oral rehydration saline (ORS) or any other medicine at home. Her urine output was normal. She had also vomited for a few episodes, which contained only food. Immunization was yet to be initiated.

On query, her mother mentioned that she was on irregular antenatal care, but her antenatal period was uneventful. The child was delivered by Caesarean section at term with a birth weight of 2.5 kg. She cried immediately after birth. No significant postnatal illness was observed.

The child was given breast milk immediately after birth. She was discharged five days after birth and at home she received mostly formula milk with occasional breastfeeding.

This child is the 2^nd^ issue of non-consanguineous parents. Their 1^st^ issue was a female child who died approximately two months after birth, five years back. According to the mother, the child was delivered by Caesarean section and was born healthy but suffered from gradual weight loss. Due to significant economic hardship, the parents were unable to provide adequate healthcare and the child ultimately died at home without a confirmed diagnosis. While this raises the possibility of a genetic etiology, no conclusive evidence is available.

The child’s father was a rickshaw puller and the mother was an embroidery worker. They lived in a brick-built house, consumed boiled tap water but shared the kitchen and toilet facilities with two other families.

## Management

Upon arrival at icddr,b, the child showed features of dehydration. Her vital signs were as follows: pulse rate of 164 beats per minute, respiratory rate of 42 breaths per minute, blood pressure of 90/50 mmHg and a body temperature of 37.5°C. Anthropometric assessment revealed a weight of 2.0 kg and a length of 44 cm, corresponding to a weight-for-age Z-score (WAZ) of −5.74, indicative of severe malnutrition. Correction of dehydration was promptly initiated with glucose-based ORS at 10 mL/kg/hour in 2 hours, followed by 5 mL/kg/hour in the next 8 hours. Nutritional support was provided with infant formula at an amount of 120 mL/kg/day. Intravenous antibiotics (Ampicillin and Gentamicin) and micronutrients were administered according to the WHO management protocol for severely malnourished children.

On the following day, the child was transferred to the intensive care unit (ICU) due to the development of sclerema. She was transfused whole human blood at a rate of 10 mL/kg over a period of 3–4 hours. The antibiotics were changed to intravenous Ceftazidime and Amikacin. Despite these interventions, her clinical condition deteriorated and on the fourth day following ICU transfer (22 August 2021), she developed fluid refractory shock. To combat shock, an additional unit of whole human blood was transfused. However, her blood pressure remained critically low at 60/30 mmHg (mean arterial pressure of 40 mmHg) even at the end of transfusion, necessitating the initiation of intravenous adrenaline infusion, the dose of which was gradually titrated up to achieve optimum mean arterial pressure (≥ 50 mmHg). During this time the child was kept NPO (nothing per oral) and intravenous fluid ration was started at a dose of 3 mL/kg/hour. She was kept warm by covering with blankets and her total daily intake and output were strictly monitored.

Approximately 12 hours after the stabilization of blood pressure, the inotrope was gradually de-escalated and finally stopped on August 25, 2021. After 11 days of close monitoring and continuous care in the ICU her condition gradually improved and she was transferred to the ward.

After the resolution of her acute clinical condition, she was transferred to the nutritional rehabilitation unit for the management of malnutrition on September 5, 2021 (20^th^ day of hospitalization). On that day, her body weight was 1.96 kg and her weight-for-age Z-score (WAZ) was −5.5. Her dietary volume was gradually increased to 150 mL/kg per day. Her vital signs and anthropometry were assessed daily. But despite having a good appetite, her weight gradually decreased. To address this unexplained weight loss, she was screened for pulmonary tuberculosis, TORCH infection (toxoplasmosis, rubella, cytomegalovirus, and herpes simplex), and HIV (Table 1). However, her serum electrolytes showed persistent normal anion gap metabolic acidosis with hyperchloremia. Urine strip test revealed acidic urine. The urine calcium-creatinine ratio increased. As the child’s urine pH remained between 5.0 and 5.5 and clinical findings suggested proximal RTA, ammonium chloride loading test was not performed. Literatures indicate that this test is the gold standard for diagnosing distal RTA. It is unsuitable for detecting proximal RTA or distinguishing it from normal renal function [3, 4, 14].

**Table 1:** Laboratory data of the child.

Variable		Reference range (pediatric)	18 August 2021	23 August 2021	13 September 2021
Hemoglobin (gm/dL)		12.0–15.5	10.0	11.3	8.6
Total White-Blood cell Count (WBC) (per μL)		7.3–16.7	5.99	1.66	8.97
Differential count of WBC (%)	Neutrophils	40–75	35	67.2	50.7
	Band	−	22	4	−
	Lymphocytes	8–68	18	23	41.9
Platelet count (per μL)		100 000–400 000	163 000	46 000	
Calcium (mmol/L)		2.25–2.75		2.29	
Magnesium (mmol/L)		0.77–1.03		0.85	
C-reactive protein (mg/dl)		<0.5		6.21	
Procalcitonin (ng/ml)		<0.1		0.87	
Plasma lactate (mmol/L)		0.5–2.2		2.02	
Arterial blood gas analysis	pH			7.26	
	PO_2_ (mmHg)			70.4	
	PCO2 (mmHg)			26.1	
	HCO_3_ (mmol/L)			11.8	
Dengue NS1 Antigen				Negative	
Blood for culture & sensitivity				*Klebsiella pneumoniae*	
Urine for culture & sensitivity				*E. coli* Streptococcus spec.	
Urine routine and microscopic examination					
			On 13 September, 2021	On 16 September, 2021	On 19 September, 2021
Appearance			Strongly turbid	Clear	Clear
pH			5.5	5.5	5.0
Protein			2+	Negative	Trace
Glucose			+	Negative	Negative
Nitrite			Negative	Negative	Negative
Leukocyte esterase			Increased	Negative	Negative
RBC (/HPF)			1–3	1–3	1–3
Pus cells (/HPF)			50–60	0–1	3–5
Other relevant investigations					
Peripheral blood film		Moderate normocytic anemia with neutrophilic leucocytosis (7 September 2021)			
Gene Xpert Ultra for MTB		MTB not detected (12 September 2021)			
TORCH panel test		IgG positive for Toxoplasma, Rubella and Cytomegalovirus (15 September 2021)			
Urine Calcium/Creatinine ratio		U. Creatinine: 930.8 micromol/L, U. Calcium: 5.502 mmol/L Ratio: 5.91(17 September 2021)			
Ultrasonogram of whole abdomen		No feature of nephrocalcinosis (19 September 2021)			

**Figure 1:**
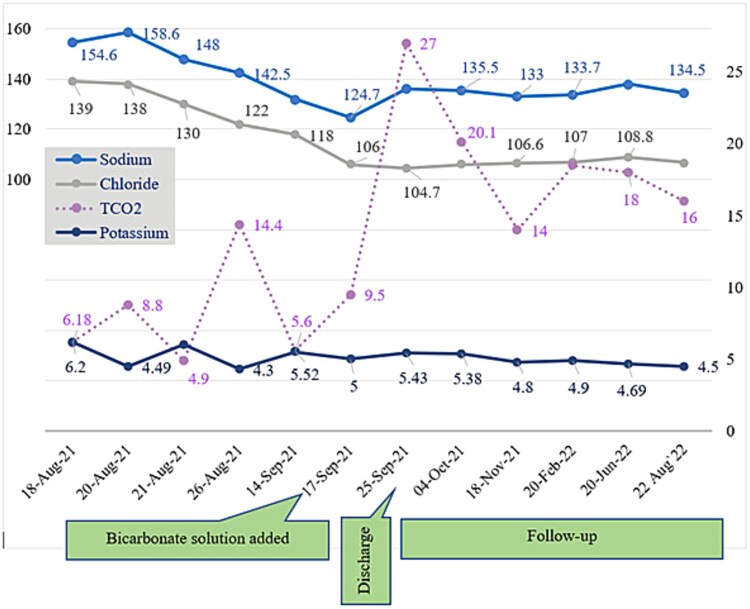
Distribution of electrolyte throughout the course in hospital and during follow up. Sustained improvement was observed in serum HCO_3_- with supplementation of bicarbonate solution.

From September 19, 2021, (on day 30 of the hospital stay), oral bicarbonate therapy was started to manage renal tubular acidosis along with potassium, calcium, and vitamin D supplementation. On that day, her weight was 1.5 kg. Oral antibiotics were administered for urinary tract infection. Subsequently, she started gaining weight (Fig. 1). On the 38^th^ day, her weight was 1.77 kg, she was clinically stable and anthropometry improved. She was discharged on 27^th^ September, 42 days after the hospital stay. On discharge she was advised to continue oral bicarbonate, potassium, calcium, and vitamin D supplementation, along with other micronutrients. Follow-up visits were scheduled to monitor the treatment response and any adverse effects.

**Figure 2:**
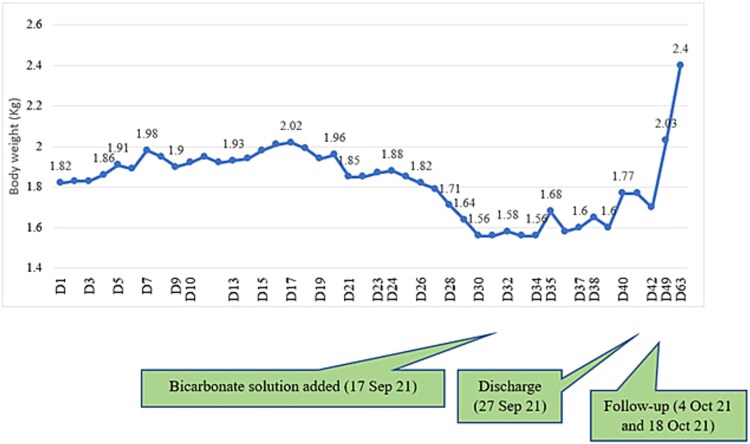
Failure to thrive during the initial phase of in-hospital stay and subsequent improvement with bicarbonate solution supplementation.

## Diagnosis

On the basis of the child’s clinical features and laboratory investigation findings, it was concluded that this child was a case of proximal renal tubular acidosis with severe malnutrition with septic shock (resolved) with sclerema (resolved) with acute kidney injury (resolved) with urinary tract infection (resolved) with acute watery diarrhoea (resolved).

## Follow up

The child was initially followed up monthly and thereafter 2–3 monthly. Her weight gain was sustained and metabolic acidosis resolved gradually. The urine strip test also showed a pattern of change from low to normal pH.

## Discussion

Proximal renal tubular acidosis (pRTA) is characterized by hyperchloremic metabolic acidosis resulting from the reduced ability of the proximal tubule to reabsorb bicarbonate. Normally, 85–90% of bicarbonate filtered through the glomerulus is reabsorbed at the proximal tubule, and the rest is reabsorbed at the distal tubule [2, 4, 11]. In pRTA, impaired bicarbonate reabsorption leads to osmotic diuresis, increased flow rate to the distal tubule and increased potassium excretion, leading to hypokalaemia [2, 4, 11].

In 2008, five members of a family were reported to be affected by a pure or isolated form of pRTA, and the only physical finding in all of them was short stature [15]. The child in this case had failure to thrive and mild hypokalemia, but no urinary abnormality except for acidosis, so she possibly had isolated pRTA not associated with Fanconi syndrome (since Fanconi syndrome involves abnormal excretion of amino acids, glucose, phosphorus and uric acid in urine).

Over the years, a number of cases of renal tubular acidosis have been reported from Bangladesh and its nearby countries with similar settings [16–22]. A study published in the *Nature Genetics* in 1999 first reported the association between rare mutations in the *SLC*4A4 gene in autosomal recessive condition and the development of isolated pRTA [2, 23]. This gene encodes the sodium bicarbonate cotransporter. Isolated pRTA resulted from such genetic mutation is permanent and also presents with ocular conditions such as glaucoma, cataract, band keratopathy and may even progress to blindness [2, 23]. However, this case is unique because of the following reasons: (a) it depicts a transient variety of isolated proximal RTA and (b) the child in this case recovered without progressing to any complication such as rickets, renal stones or ocular manifestations, unlike the other cases. Thus, this case propagates a strong message that early diagnosis and proper treatment of renal tubular acidosis can lead to the best possible outcome.

## Strength and limitation

The child was followed-up for a long period with regular monitoring of growth, assessment of bony deformities and renal function. However, no genetic or molecular study could be done.

## Learning points

▪ RTA often presents with failure to thrive within the first year of life. Children showing growth failure along with polyuria, excessive thirst, or rickets-like features should be evaluated for RTA.
▪ Loss of nutrients and electrolytes through the renal tubules, along with reduced bone growth from acidosis, leads to growth failure.
▪ The persistence of normal anion gap metabolic acidosis, hyperchloremia, and acidic urine pH after resolution of diarrhea is suggestive of proximal RTA.
▪ Long-term follow-up is needed to monitor growth, bone deformities, urinary infections, and renal function, along with adjusting bicarbonate supplementation doses.
▪ Family members should undergo a comprehensive clinical and metabolic evaluation. If available, molecular testing can help identify the genetic defect and define its phenotype.

## CARE checklist (2016) statement

The authors have read the CARE checklist (2016) and the manuscript has been prepared and revised accordingly.

## Informed consent statement

Written informed consent was obtained from the patient’s guardian.

## Supplementary Material

Supplementary_file__photo_omaf294
