# Supplementary figures and images for: Severe growth faltering and persistent hyperchloremic metabolic acidosis as early clues of renal tubular acidosis in a neonate: a case report
Source: Oxf Med Case Reports. 2026 Feb 18;2026(2):omaf294. doi: 10.1093/omcr/omaf294 (PMC12915056; doi:10.1093/omcr/omaf294)

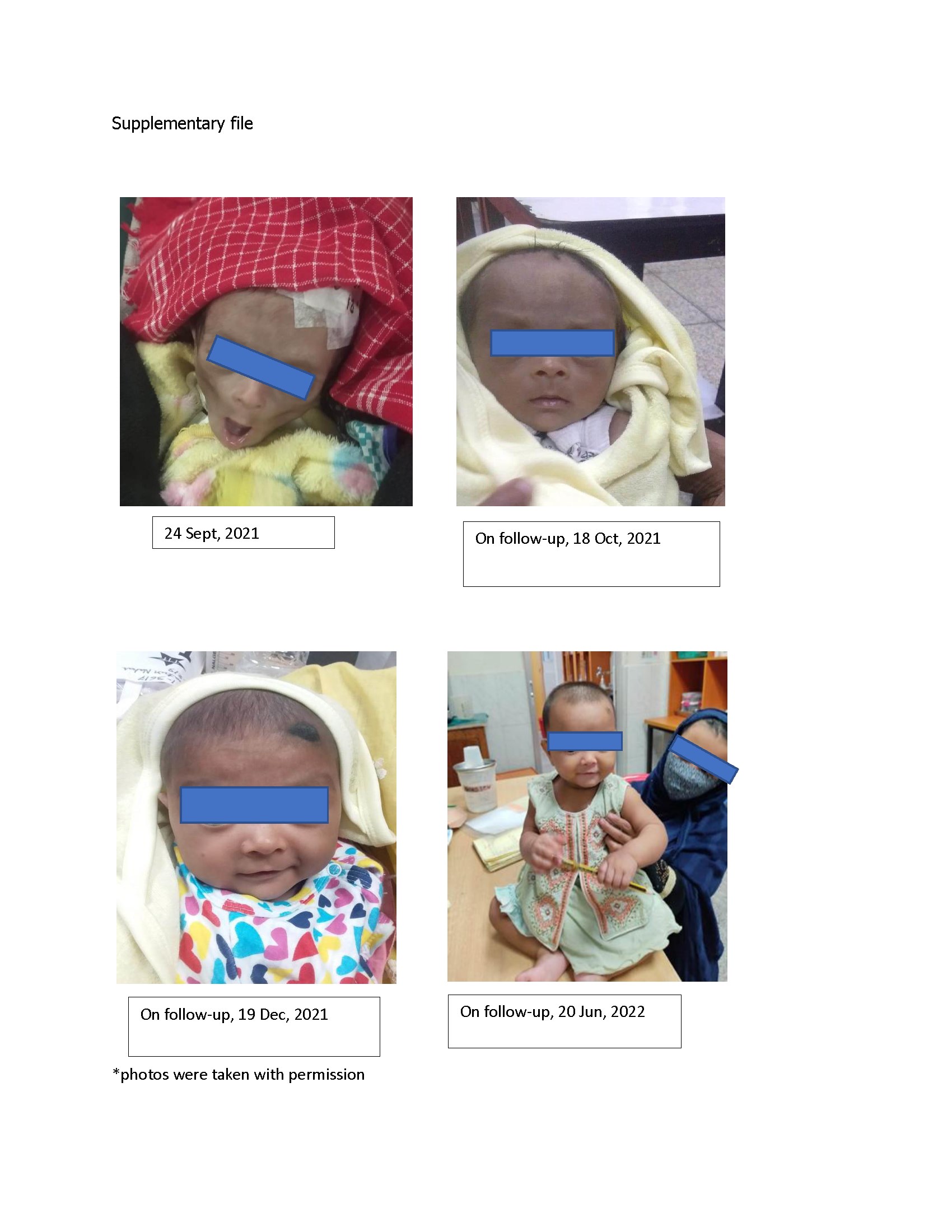

Supplement: Supplementary_file__photo_omaf294 [file supplementary_file__photo_omaf294.jpeg]
